# A Framework for Measuring Neighborhood Walkability for Older Adults—A Delphi Consensus Study

**DOI:** 10.1007/s11524-024-00910-7

**Published:** 2024-09-03

**Authors:** Daniela Koller, Malte Bödeker, Ulrike Dapp, Eva Grill, Judith Fuchs, Werner Maier, Ralf Strobl

**Affiliations:** 1 Institute of Medical Information Processing, Biometry, and Epidemiology, Faculty of Medicine, LMU Munich, Munich, Germany; 2 Federal Centre for Health Education, Cologne, Germany; 3 Geriatrics Centre, Scientific Department at the University of Hamburg, Albertinen-Haus, Hamburg, Germany; 4 German Center for Vertigo and Balance Disorders, LMU University Hospital, Munich, Germany; 5 Department of Epidemiology and Health Monitoring, Robert Koch Institute, Berlin, Germany

**Keywords:** Walkability, Functioning, Age, Neighborhood, Delphi, Qualitative research

## Abstract

While mobility in older age is of crucial importance for health and well-being, it is worth noting that currently, there is no German language framework for measuring walkability for older adults that also considers the functional status of a person. Therefore, we combined the results of an expert workshop, a literature review, and a Delphi consensus survey. Through this, we identified and rated indicators relevant for walkability for older adults, additionally focusing on their functional status. The expert workshop and the review led to an extensive list of potential indicators, which we hope will be useful in future research. Those indicators were then adapted and rated in a three-stage Delphi expert survey. A fourth additional Delphi round was conducted to assess the relevance of each indicator for the different frailty levels, namely “robust,” “pre-frail,” and “frail.” Between 20 and 28 experts participated in each round of the Delphi survey. The Delphi process resulted in a list of 72 indicators deemed relevant for walkability in older age groups, grouped into three main categories: “Built environment and transport infrastructure,” “Accessibility and meeting places,” and “Attractiveness and sense of security.” For 35 of those indicators, it was suggested that functional status should be additionally considered. This framework represents a significant step forward in comprehensively covering indicators for subjective and objective walkability in older age, while also incorporating aspects of functioning relevant to older adults. It would be beneficial to test and apply the indicator set in a community setting.

## Introduction

In the context of demographic change, the number of older adults is rising and will continue to increase. To ensure them, a long, independent, and healthy life is a main topic in public health. Physical activity plays an important role for successful aging and social participation [1,2]. Parallel to demographic change there is a trend towards urbanization. Both trends together can be especially challenging for cities to adapt their built environment towards their aging population [3].

The ongoing urbanization has brought specific attention to the importance of walkability, which refers to the ease and safety of walking within a specific environment. High walkability can promote physical activity and social participation if it addresses the requirements of the people within their daily lives. For older adults, this may be a key indicator of longer independent living and of aging in place [4–8].

The degree of walkability in a neighborhood can be assessed by a variety of indicators, which have been adapted to specific settings or populations. These include, for example, indicators for urban and rural environments, as well as for children [9,10]. So far, walkability instruments suited to the general population (see [11] for an overview) have been used for measuring walkability in the aging population. Those instrument might however not fully capture the challenges and needs of an aging population as they might lack indicators specifically addressing needs of older adults, especially with respect to physical restrictions due to functional decline. This necessitates the use of a better targeted evaluation approach.

The assessment of walkability in neighborhoods for older adults necessitates the consideration of several key factors, which can be broadly divided into objective and subjective aspects of walkability. Objective walkability comprises elements of urban planning [12] or how easy and fast essential destinations can be reached [13]. In particular, walkable neighborhoods are characterized by a high population density (that are usually associated with a higher number of destinations/amenities, higher potential for social interaction, or more local infrastructure [13]), a wide range of local services, a dense network of footpaths, and traffic lights that are designed to accommodate slower walking speeds, ramps with low gradients, or resting areas. In addition to these features of the pedestrian infrastructure, destinations such as parks or essential infrastructure such as public transit stops, retail, pharmacies, or doctors’ offices must also be present [14].

Subjective walkability encompasses the individual’s perception of their neighborhood, particularly with regard to safety and aesthetics. A neighborhood may be perceived as safe if pedestrians feel protected from motorized traffic or potential crime. Neighborhoods may be considered attractive for walking if they are interesting, aesthetically pleasing, or simply populated and well-lit. Additionally, in the context of global warming, the presence of shade, for instance, provided by a tree canopy, [15] or protection from rain downpours, plays an increasingly significant role in shaping perceptions of safety [16–19]. Of note, there is a strong interaction between features of the objective built environment and the subjective perception of walkability.

While the positive effect of a walkable environment on older adults’ health has been shown [4,5,7,20–30], even for persons who already experience mobility restrictions [23,30], the relationship between age and behavior in the built environment is more complex [22]. Specifically, walkability frameworks for the general population should consider potential facilitators and barriers of mobility of older adults. Arguably, among these, functioning, disability, and frailty are the most salient factors [31,32].

The concepts of functioning and disability are often defined as the complex interactions between physical functions, activities of daily living, social participation, and personal and environmental context [33]. It is crucial to capture the full range of human experiences of functioning in order to align the concept of walkability within the context of older age.

The objective of the present study is to construct a framework for walkability in older age that includes relevant indicators of the neighborhood, with a specific focus on the functioning of an older population. Two lists of indicators are proposed: a comprehensive assessment list and a minimal standard list, which focus on salient indicators that are easy to apply in an urban neighborhood setting.

## Methods

The aim of this study was to establish (a) a “minimal standard” and (b) a “comprehensive assessment” indicator set. The minimal standard set is intended to be a short list of indicators and aims to serve as the minimal standard for assessing walkability in older people, e.g., for urban planning or communication with stakeholders. The comprehensive assessment aims to serve as a basis for full assessment and documentation when time and resources allow a comprehensive assessment, e.g., for research purposes.

### Overview of multistage methodology

**Fig. 1:**
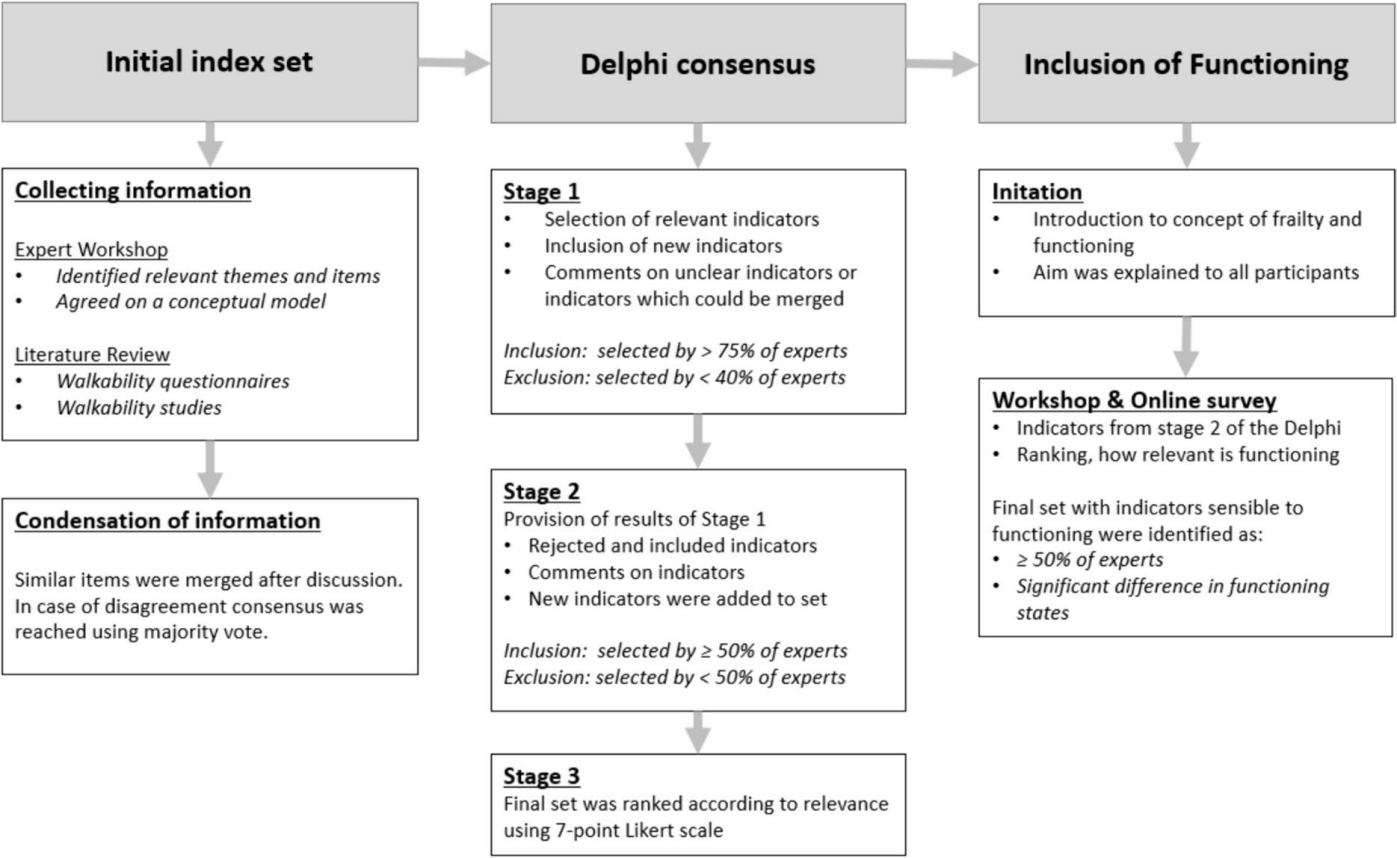
Flowchart representing the steps for developing a framework for assessing neighborhood walkability for older adults

To capture relevant indicators of walkability in older people, a multistage methodology was applied consisting of an expert workshop and an extensive literature review. Results of both were then combined and ranked in a 3-stage Delphi consensus process. We give an overview of each of the steps in the following Fig. 1.

### Preparatory Expert Workshop

An expert workshop on walkability in old age organized by the working groups “Epidemiology of Aging” and “Health Geography” that are accredited in the German Society for Epidemiology (DGEpi) was conducted. All members of the DGEpi and the interest mailing list of both working groups (irrespective of DGEpi membership status) were invited. The invitation was further circulated through other networks and cooperations, including experts in the fields of urban planning, aging, and health geography. Seventeen participants with different backgrounds took part in the expert workshop: six were experts in aging, five were experts in walkability and health geography, two were public health experts, and four were practitioners working for public service. Using group-based methods, aspects of conceptualization, operationalization, and measurement of walkability in old age were discussed and elaborated. This was done by means of keynote speeches and project presentations followed by discussion rounds. The participants agreed on a conceptual basic model and identified a list of preliminary indicators based on the presented information and the results of the group discussions.

### Literature Review

A literature review identified already established indicators for assessing general walkability. It focused on questionnaires available in the German language. RS and DK independently conducted literature searches via: PubMed, source review of known questionnaires and item sets, search of project sites, and gray literature. The search was continuous and not systematic, search terms were (“walkability” OR “walk-friendly” OR “physical activity”) AND (“age” or “older adults”) AND (“index” OR “framework” OR “instrument”). Search results were extended by including forward and backward citations. The results were jointly reviewed and integrated into the indicator set after consensus building. The result was finally discussed with UD and MB and jointly decided. The literature search was last updated before the start of the Delphi consensus study on September 1st, 2020.

### Delphi Consensus Study (Three Stages)

The decision-making process required input and perspectives from a diverse group of experts which can be best integrated by the use of a Delphi method. By anonymizing responses and facilitating multiple rounds of feedback, the Delphi method helps to reduce the influence of dominant voices and allows participants to revise their opinions based on the collective insights shared during the process [34,35]. This iterative approach fosters a refined consensus leading to well-informed and thoughtful decisions in areas where a comprehensive understanding is crucial. The aim of the Delphi survey was to identify missing indicators and to select a set of relevant indicators of walkability in older people.

The indicators identified through the expert workshop and literature review were structured as proposed by the experts of the workshop. In case an indicator did not fit, the structure was adapted after discussion among the study group for an online Delphi consensus survey. The experts were invited for the Delphi survey if they (a) participated in the expert workshop, (b) were recommended by one of the participating experts/forwarded the invitation, (c) were identified as author of the identified literature, and (d) were members of the Working groups Epidemiology of Aging or Health Geography in the German Society for Epidemiology or the Medical Geography Study Group in the German Geographic Society. The invitation for the Delphi survey was sent to 60 identified experts, by e-mail providing a description of the project and the link to the survey. All experts were invited to forward the invitation to interested colleagues.

Based on the experience from previously conducted Delphi surveys, the survey was designed in three stages [36,37] (see Fig. 1).

I. In the first stage, the experts were asked for each indicator whether they thought it was relevant to walkability in older age. In addition, comments could be made on each indicator as well as on each category and additional indicators could be mentioned. In accordance to previous Delphi surveys, indicators with an agreement of more than 75% were included in the final set. If the agreement was between 40 and 75%, the indicator was included in the second stage; if the agreement was below 40%, the indicator was rejected. We chose the decision thresholds based on previous experiences [36,37]. Comments and suggestions for new indicators were independently reviewed by DK and RS and implemented by consensus for the second stage. In case of ambiguity, additional UD was added for consensus.
II. In the second stage, comments were evaluated, and indicators were reformulated or joined accordingly. The proposed new indicators as well as the indicators that were not clearly accepted or rejected in the first stage (relevance to walkability in older age yes/no) were included in the Delphi survey. In this stage, a 50% cut-off applied to acceptance or rejection into the final indicator set.
III. In the third stage, comments were evaluated, and indicators were reformulated or merged accordingly. The final indicator set was evaluated according to relevance. For this purpose, the indicators were rated by the experts on a 7-point Likert scale as to how relevant the indicator is to walkability in older age (including answering categories were ranked from 1 (less important) to 7 (needs to be included)). In addition, the top categories and the subcategories were ranked according to their relevance. No indicators were rejected at this stage; however, through the ranking created here, cut-off values can be defined in the practical implementation of the indicator set.

All data was anonymously recorded in each of the three stages; thus, no information on age, gender, or years of experience was provided. The survey took place online from September 2020 to January 2021.

### Expert Round for Functioning

Discussions and comments pointed out that walkability should not only take biological age into account, but also the domain functional ability. To include this domain, an additional expert panel was conducted consisting of the existing expert panel and additional experts on aging and functioning. All experts received an introduction to the concept of functioning as a geriatric functional continuum modified from theoretic models [38,39]. These theoretic models were accompanied by pictures and empirical data on functioning to demonstrate older persons’ different physical functional levels from robustness (independence) to disability (dependence). On this continuum, a result of the impact of multiple system reduction in reserve capacity is conceptualized as frailty, a measurable syndrome consisting of weakness, poor endurance, reduced physical activity, slow gait, and unintentional weight loss [40,41].

All experts rated the relevance of the indicators for walkability according to the three different frailty phenotypes “robust,” “prefrail,” or “frail.” Robust adults are defined as having a high functional ability; for the pre-frail, the resources are declining with increasing risks for physical vulnerability, whereas the frail population is considered frail but not as disabled or in need of nursing care.

Experts ranked the relevance on a 5-point Likert scale from 1 (little relevance) to 5 (highly relevant). If functioning was not considered relevant the answer category “same for all” could be chosen. In order to be included in the final sets, two criteria must have been met for each indicator: ≥ 50% of the experts stated that functioning should be considered and the difference in the rating between “robust,” “pre-frail,” and “frail” had to be statistically significant. Kruskal–Wallis tests were applied for testing for these differences.

All steps were carried out in accordance with the Declaration of Helsinki. There is a lack of valid reporting guidelines for Delphi studies; we did however work in accordance with the available guidance [35]. This study is based on an anonymized qualitative study, and an ethics approval was therefore not necessary. The preparatory expert workshop took place as a face-to-face meeting in Munich in July 2018 (supported by the German Society for Epidemiology, DGEpi). The Delphi survey was conducted online using the SoSciSurvey tool (version 3.1.06) [42]. The Delphi analyses took place with R (version 4.2.2) [43]. The Ethics Committee of the Medical Faculty of the LMU Munich evaluated this study and issued a waiver (4–0236 KB).

## Results

### Preparatory Expert Workshop and Literature Review

A total of 17 experts participated in the preparatory workshop.

Through the expert workshop and the literature review, 152 indicators were identified. After the first Delphi round, they were organized into four main categories (following the dimensions identified in the workshop) and 14 subcategories (see Table 1).

**Table 1:** Main and subcategories after the first Delphi round

Main category	Subcategory
Built environment and transport infrastructure	• Road safety • Pedestrian infrastructure • Bicycle infrastructure
Accessibility and meeting places	• Parks • Public transport • Toilets • General supply, including medical care • Availability of exercise possibilities • Social encounters
Attractiveness and sense of security	• Crime (e.g., drug sales, burglaries) • Subjective security • Pollution • General attractiveness
Others	

### Delphi Consensus Survey

**Fig. 2:**
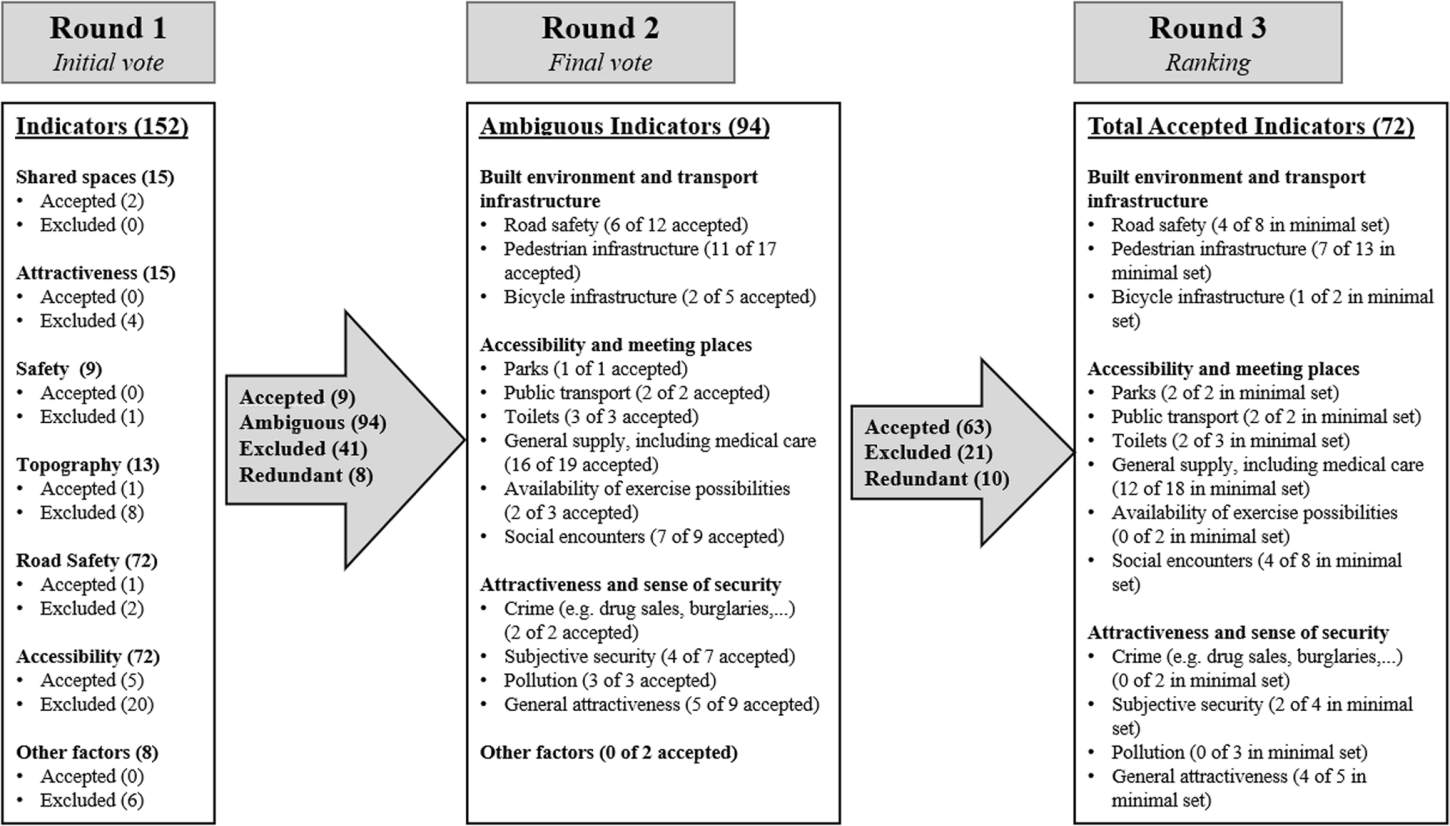
Content map of the Delphi survey

The results of the three rounds of the Delphi survey are visualized in a content map in Fig. 2.

In the first round, 28 experts participated. Nine items were accepted by majority, and 38 indicators were excluded according to our set methods. Based on the comments, 12 indicators were reformulated, 10 were reworded, and 6 indicators were deleted due to redundancies. Redundancies occurred since we included similarly phrased items from different instruments. The other indicators were re-evaluated in the second stage. Likewise, the structure of the items was adjusted.

In the second stage, 23 experts participated. Twenty-one indicators were rejected, 63 were accepted, and 10 indicators were adapted joined according to the commentaries.

In the third stage, 20 experts participated. The remaining 72 indicators were rated within the categories by a 7-point Likert scale. In the next step, the main categories and the subcategories were ranked by relevance to walkability in older age.

The indicators were sorted by average score per subcategory. All 72 indicators are included in the final indicator set. For the potential practical application of the indicator set, it is recommended to define category-specific cut-off values to include the most relevant indicators (see Tables 2, 3, and 4). For this, we suggest all indicators with a median rank of 6 or higher to be considered as candidates for the minimal standard set. A median rank of 6 or more corresponds to above 50% of the experts assessed the indicator as relevant. All considered indicators, if they are included in the minimal standard set and if they are considered functioning sensitive can be found in Tables 2, 3, and 4.

**Table 2:** Indicators of the main category “Built environment and transport infrastructure” included in the comprehensive list (*n* = 23). Vote I and II indicate the stage of the process at which an indicator was included in the list. The indicators marked with X were included in the minimal standard set using a ranking cut-off of 6. An * marks a reverse association of functioning and relevance

Description	Vote	Minimal standard set	Functioning should be considered
***Road safety (n* = *8)***			
Traffic calming measures, such as speed limits	I		
Separation of pedestrian/bicycle path/road	I	X	
Crossing busy streets is made easier (pedestrian lights, zebra crossings, pedestrian islands)	II	X	X
Traffic light switching (e.g., green phase duration)	II	X	X
Free view (good visible paths, etc.)	II		X
There are significant obstacles in my residential environment that make it difficult to get from A to B (for example: motorways, railway lines, rivers)	II		
Conflicts of use about the available space (parking/stopping cars, delivery traffic on the sidewalk, cyclists on sidewalks)	II	X	
Handrails in parks, underpasses, etc	II		X
***Pedestrian infrastructure (n* = *13)***			
Seating possibilities	I	X	X
I can do a lot of things on foot from my apartment	I	X	X
Seating options are shaded	II		X
Ground conditions (e.g., surface quality, no potholes)	II	X	X
Wide footpaths	II		X
Stairs have railings/handrails	II		X
Design alternatives without stairs and steps (e.g., ramp)	II		X
There are many different ways to get from A to B	II		X
Possibility to walk in your living area	II	X	X
Poor quality of the sidewalks (inadequate winter service, poor lighting, disruptive factors such as bicycles, garbage cans, etc.)	II	X	X
Lowered border stones	II	X	X
Lighting	II	X	
Trees provide shade on the sidewalks in my neighborhood	II		X
***Bicycle infrastructure (n* = *2)***			
Possibility to ride a bike in your living area	II	X	X*
Lighting	II		X*

**Table 3:** Indicators of the main category “Accessibility and meeting places” included in the comprehensive list (n = 35). Vote I and II indicate the stage of the process at which an indicator was included in the list. The indicators marked with X were included in the minimal standard set using a ranking cut-off of 6

Description	Vote	Minimal standard set	Functioning should be considered
***Parks (n* = *2)***			
Seating	I	X	X
Green spaces	II	X	
***Public transport (n* = *2)***			
Accessibility of public transport in your living area (bus or train station)	II	X	
The stops/stations are well equipped (e.g. seating, roofing, level access, lighting)	II	X	X
***Toilets (n* = *3)***			
There are enough toilets (public and publicly accessible)	II	X	
Toilets (public and publicly accessible) are easy to find	II		
Toilets (public and open to the public) are in acceptable condition	II	X	
***General supply, including medical care (n* = *18)***			
Health care (density of doctors and specialists, physiotherapy, etc.)	I	X	X
Bakery, butcher, or similar	I	X	X
Mixed use of housing, services, and retail in the town center	II	X	
Supermarket	II	X	X
Fruit and vegetable shop	II	X	
Postal service	II	X	
Public library	II		
Café	II	X	X
Bank/credit institution	II	X	
Restaurant	II		X
Pharmacy	II	X	X
Barber	II	X	X
I can do most of the shopping in shops close to where I live	II	X	
Shops are an easy walk from my apartment	II	X	X
Weekly market	II		X
Citizens’ office, municipal office	II		X
Cemetery	II		X
Cultural facilities/offers (theater, exhibitions, etc.)	II		
***Availability of exercise possibilities (n* = *2)***			
Play and movement equipment (e.g. boules court, large field chess, fitness equipment)	II		X
Accessible lawns for games and sports	II		X
***Social encounters (n* = *8)***			
Neighborhood association, meeting points/town center	I		
Senior centers	II		
Number of friends and acquaintances in your living area	II	X	X
Number of relatives in your living area	II		X
Enabling encounters between generations	II	X	
Areas for different needs, e.g., rest and activity	II	X	
Religious site (church, synagogue, house of prayer, etc.)	II		
Seating options enable communication/meeting points (e.g., seating groups)	II	X	X

**Table 4:** Indicators of the main category “Attractiveness and sense of security” included in the comprehensive list (*n* = 14). Vote I and II indicate the stage of the process at which an indicator was included in the list. The indicators marked with X were included in the minimal standard set using a ranking cut-off of 6

Description	Vote	Minimal standard set	Functioning should be considered
***Crime (e.g., drug sales, burglaries) (n* = *2)***			
The crime rate in my living area is high	II		
Crime in my neighborhood makes walks unsafe during the day	II		
***Subjective security (n* = *4)***			
I see and speak to others when I walk through my living area	II	X	
Feeling protected from crime	II		
Social insecurity in underpasses, dead corners and on poorly lit sidewalks	II	X	
Free view (visibility, e.g., from underpasses)	II		
***Pollution (n* = *3)***			
Clean (semi-)public spaces	II		
Enough rubbish bins	II		
Dog waste bags supply	II		
***General attractiveness (n* = *5)***			
Poor quality of stay due to heavy traffic	II	X	
Air quality (e.g., poor quality of stay due to exhaust fumes)	II	X	
There are many interesting things to see while walking around my living area	II	X	
Nature that is beautiful to look at (such as landscapes, viewpoints)	II		
Poor quality of stay due to noise	II	X	

### Expert Round for Functioning

A total of 20 experts participated in rating the 72 indicators identified by the first Delphi consensus survey. For 54 indicators, at least half of the experts stated that functioning should be considered. For 58 indictors, the ratings for the relevance to walkability were significantly different for the groups “robust,” “pre-frail,” and “frail.” This resulted in a total of 35 indicators for which functioning should be considered. Most functioning sensitive indicators are in the category pedestrian infrastructure and general supply, including medical care. Air pollution, crime, and general attractiveness were considered as relevant irrespective of the individual functioning status.

## Discussion

“Successful aging” is a multidimensional concept and contains both biomedical aspects and psychological aspects, such as the ability to actively engage in life, autonomy, and social participation [33] which are all related to the direct neighborhood [4–7]. Walkability is a main factor for this; however, there are no comprehensive measurement tools for walkability in older age in Germany, and very few internationally [44,45].

This is the first framework that comprehensively covers indicators for subjective and objective walkability in older age and at the same time incorporates aspects of functioning relevant to older adults. Thus, our study has the potential to fill a gap in walkability research.

We based our Delphi study on a review of the existing literature. Included studies mostly highlighted selected single aspects of walkability. In a Belgian study, for example, walkability for the older population was measured on the basis of objective land use characteristics, such as population density, connectivity and mix of uses [29,46]. A study from the Netherlands included an assessment of the density of green spaces and footpaths [26].

Arguably, out study is the first to take functioning into account. Our Delphi survey of experts found that pedestrian infrastructure and the ease of access to general amenities, including medical care, were cited as the most significant factors. This is supported by the finding that an inferior street connectivity was related to increased frailty of the older residents [31]. Recent evidence from the Longitudinal Urban Cohort Ageing Study (LUCAS) found that an urban environment that promotes physical activity might prevent the onset of functional decline and stresses the importance of early detection of pre-clinical stages of frailty in the context of the urban environment the individual lives in [40,47]. They also found that the functional level had an impact on an individuals’ ability to reach essential places such as shops, post offices, and GP practices, but also cultural or recreational facilities or public parks, lakes, or rivers [47,48].

The importance of subjective walkability based on frailty was shown by a study from Japan. They found that pre-frail or frail adults perceived not only walking and cycling facilities as poorer than robust participants, but also the aesthetic appeal and safety from crime in their neighborhood [32].

While sociodemographic status was not part of our indicator set, a study from Belgium disentangled the complex association of financial assets, neighborhood, and functioning [28]. Arguably, neighborhood design regarding walkability will have a positive effect on functioning, but persons with better financial resources also tend to live in healthier environments.

Also, policy background of aging in the urban environment has to be taken into account. As countries and regions widely differ regarding their healthcare planning policies and programs to cope with the challenges of aging in health, international results are not easily transferable ^49^.

In order to meet the needs of the older population in particular, our comprehensive framework gives a set of indicators that now encourages measuring walkability of a neighborhood specifically for older people, while specifically considering their functional status. This indicator set must now be operationalized in the form of a questionnaire and tested for applicability.

Through this, municipalities could be provided with an instrument through which individual residential areas can be tested for walkability for their older inhabitants and which can be used in potential urban renewal processes in order to make the residential environment walk-friendly for all residents. The next steps will be to test and validate our indicator sets in test cities, including senior residents as well as urban planning and the neighborhood multiplicators. This will include structured neighborhood walks and organized discussions with the involved groups in order to create a tool that is usable to enhance neighborhood walkability for older adults in an urban setting.

## Data Availability

This study is based on qualitative research. Data are available upon request.
